# Distinct regulatory pathways contribute to dynamic CHH methylation patterns in transposable elements throughout *Arabidopsis* embryogenesis

**DOI:** 10.3389/fpls.2023.1204279

**Published:** 2023-06-08

**Authors:** Jaehoon Lee, Seunga Lee, Kyunghyuk Park, Sang-Yoon Shin, Jennifer M. Frost, Ping-Hung Hsieh, Chanseok Shin, Robert L. Fischer, Tzung-Fu Hsieh, Yeonhee Choi

**Affiliations:** ^1^ Department of Biological Sciences, Seoul National University, Seoul, Republic of Korea; ^2^ Research Center for Plant Plasticity, Seoul National University, Seoul, Republic of Korea; ^3^ Department of Agricultural Biotechnology, Seoul National University, Seoul, Republic of Korea; ^4^ Department of Plant and Microbial Biology, University of California, Berkeley, Berkeley, CA, United States; ^5^ Department of Plant and Microbial Biology, North Carolina State University, Raleigh, NC, United States; ^6^ Plants for Human Health Institute, North Carolina State University, Kannapolis, NC, United States

**Keywords:** DNA methylation, CHH methylation, transposable elements, CMT2, RdDM, embryogenesis

## Abstract

CHH methylation (mCHH) increases gradually during embryogenesis across dicotyledonous plants, indicating conserved mechanisms of targeting and conferral. Although it is suggested that methylation increase during embryogenesis enhances transposable element silencing, the detailed epigenetic pathways underlying this process remain unclear. In *Arabidopsis*, mCHH is regulated by both small RNA-dependent DNA methylation (RdDM) and RNA-independent Chromomethylase 2 (CMT2) pathways. Here, we conducted DNA methylome profiling at five stages of *Arabidopsis* embryogenesis, and classified mCHH regions into groups based on their dependency on different methylation pathways. Our analysis revealed that the gradual increase in mCHH in embryos coincided with the expansion of small RNA expression and regional mCHH spreading to nearby sites at numerous loci. We identified distinct methylation dynamics in different groups of mCHH targets, which vary according to transposon length, location, and cytosine frequency. Finally, we highlight the characteristics of transposable element loci that are targeted by different mCHH machinery, showing that short, heterochromatic TEs with lower mCHG levels are enriched in loci that switch from CMT2 regulation in leaves, to RdDM regulation during embryogenesis. Our findings highlight the interplay between the length, location, and cytosine frequency of transposons and the mCHH machinery in modulating mCHH dynamics during embryogenesis.

## Introduction

DNA methylation is crucial for regulating gene transcription, transposon silencing, and genomic imprinting in eukaryotic genomes. It also plays a critical role in development, and aberrant DNA methylation results in abnormal gene expression and developmental defects in vertebrates and plants (Deniz et al., 2019; Greenberg and Bourc'his, 2019). In addition to CG methylation, plant DNA also contains methylated cytosines in CHG and CHH contexts (Law and Jacobsen, 2010). Therefore, plants have evolved a comprehensive DNA methylation system involving multiple associated enzymes. Small RNA-directed DNA methylation (RdDM) is a representative feature of this system (Matzke and Mosher, 2014; Matzke et al., 2015). The RdDM pathway recruits DOMAINS REARRANGED METHYLTRANSFERASE 1/2 (DRM1/2) to their target sites, where *de novo* methylation occurs at all cytosine contexts (Cao and Jacobsen, 2002; Stroud et al., 2013). Symmetric CG and CHG methylation can be maintained by DNA METHYLTRANSFERSE 1 (MET1) and CHROMOMETHYLASE 3 (CMT3), respectively, on the opposite strand independently of small RNA (sRNA) once *de novo* methylation has occurred (Law and Jacobsen, 2010). For CHH methylation, in addition to DRM1/2, CMT2 also methylates CHH sites, mainly in the body of long transposable elements (TEs), independently of sRNA molecules (Zemach et al., 2013).

Transposable elements (TEs), which are mobile genetic entities, make up a significant portion of the genome across different species that vary from 12% of nematode *Caenorhabditis elegans* genome up to 85% of the maize genome, for example (Jurka et al., 2011; Chenais et al., 2012). TEs are considered as insertional mutagens and affect host genome size through amplifying TE copy number, thereby serving a major driving force of genome evolution (Lanciano and Cristofari, 2020). Although some TE insertions can lead to adaptive effects to the host genome by relocating *cis*-acting elements that influence gene expression, most TE insertions are neutral or midly detrimental to the host (Deniz et al., 2019). TEs are classified into two large groups depending on their mechanism of replication; retrotransposons (class I) which transpose *via* an RNA intermediate, and DNA transposons (class II). Retrotransposons are subdivided depending on the presence or lacking of long terminal repeat (LTR). Gypsy and Copia superfamilies belong to LTR retrotransposons whereas LINEs and SINEs are non-LTR retrotransposons. Major *Arabidopsis* DNA transposons (Tn) are subdivided into two groups depending on the presence or lacking of terminal inverted repeats (TIR). TIR Tns harbor TIR at the borders such as MuDR and En-Spm superfamily, and the non-TIR Helitron Tns are known to replicate *via* a rolling circle mechanism (Quesneville, 2020).

While DNA methylation patterns are replicated faithfully during cell division, preserving genome stability and cell lineage, they also need to be dynamically reprogrammed during development to establish new transcriptional states and cell fates. The dynamic regulation of DNA methylation is essential for animal development and reproduction. In mammals, DNA methylation is reprogrammed in each generation to erase and re-establish parental imprints for the next generation (Seki et al., 2005; Sasaki and Matsui, 2008; Seisenberger et al., 2013; Heard and Martienssen, 2014). In angiosperms, active demethylation occurs before fertilization in central and vegetative cells, adjacent companion cells of the egg and sperm, respectively (Schoft et al., 2011; Ibarra et al., 2012; Park et al., 2016; Kim et al., 2019). In *Arabidopsis*, this pre-fertilization epigenetic reconfiguration is performed by DEMETER (DME), a novel and essential DNA glycosylase that initiates the excision and removal of methylated cytosines *via* the base excision repair (BER) pathway. This process affects DNA methylation and gene transcription profiles in the endosperm (Gehring et al., 2006; Ibarra et al., 2012). Consequently, genome-wide DNA hypomethylation and gene imprinting are primarily observed in the endosperm upon fertilization in *Arabidopsis* (Gehring and Satyaki, 2017; Batista and Kohler, 2020).

While the presence of a bona fide DME ortholog in monocots remains debated, the ROS1a protein has been shown to be the functional counterpart of DME in the rice cultivar Nipponbare (Ono et al., 2012). The rice vegetative cell (VC) genome is also extensively hypomethylated compared to sperm, and this VC hypomethylation is dependent on ROS1a (Kim et al., 2019). Therefore, gamete companion cell epigenetic remodeling by DNA glycosylases is an evolutionarily conserved phenomenon in rice and *Arabidopsis*, despite their divergence over 150 million years ago. Multiple genome-wide DNA methylation studies have confirmed that the endosperm genome is hypomethylated relative to that of embryo in various plant species, including *Arabidopsis*, rice, maize, and Castro bean (Lauria et al., 2004; Gehring et al., 2009; Hsieh et al., 2009; Zemach et al., 2010; Xu et al., 2016; Gent et al., 2022; Xu et al., 2022).

Compared to the extensive epigenetic reprograming observed in gamete companion cells and endosperm, the embryo methylome, at least in the context of CG methylation, appears to remain static during development and between generations, ensuring robust transgenerational epigenetic inheritance (Hsieh et al., 2016; Bewick and Schmitz, 2017; Picard and Gehring, 2017). In contrast, dynamic and extensive post-fertilization reconfiguration of CHH methylation has been reported during embryogenesis and germination processes (Kawakatsu et al., 2016; Bouyer et al., 2017; Lin et al., 2017). This gradual increase in CHH methylation during embryogenesis is associated with bursts of sRNA accumulation during embryo development and an increase in RdDM activity, particularly during embryo maturation (Papareddy et al., 2020). One function of this increase in CHH methylation is thought to help suppress the activity of TEs and prevent their transposition, maintaining genomic integrity (Lin et al., 2017).

Although DNA methylation changes during plant embryo development have been reported in multiple plant species (Xing et al., 2015; Bouyer et al., 2017; Kawakatsu et al., 2017; Lin et al., 2017; Grover et al., 2020; Rajkumar et al., 2020), a comprehensive understanding of methylation dynamics for different TE groups or families during embryo development has not been reported. Recently, we described methods for isolating high-purity stage-specific embryos for low-input methylome library construction and data analysis (Yoo et al., 2021). In this study, we used these methods to investigate comprehensive methylome changes in developing *Arabidopsis* embryos at five stages. Our findings revealed distinct methylation dynamics for different TE classes according to their length, superfamily, genome location, and cytosine ratio. Shorter, heterochromatic TEs in embryos are prone to be newly targeted by the RdDM pathway upon fertilization. Furthermore, we discovered that many CHH-methylated regions expand into neighboring unmethylated regions during embryo maturation, accompanied by the accumulation and expansion of 24nt sRNA clusters. Overall, our study provides a detailed analysis of the dynamic changes in TE methylomes during *Arabidopsis* embryo development, shedding light on the underlying mechanisms of TE regulation and their potential roles in embryo development.

## Materials and methods

### Plant materials and growth conditions

The *Arabidopsis thaliana*, Col-*gl* (Columbia-glabrous) ecotype was used for embryo isolation. Plants were cultivated in a controlled environment room with a long photoperiod of 16 hours of light and 8 hours of darkness, at a temperature of 22°C with cool white fluorescent light (100 mmole/m^2^/s).

### Embryo isolation and construction of whole-genome bisulfite sequencing

After 24 hours of emasculation, fully matured WT ovules were pollinated with WT pollen. The pollinated plants were then incubated in the growth room until they reached a suitable stage for sampling. Arabidopsis embryos at DAP4, 5, 7, 9, and 12, corresponding to the globular, heart, torpedo, bending torpedo, and mature green stage embryos, respectively, were dissected from seeds using various methods specific to each stage. Globular embryos were obtained by gentle grinding the seeds with a pestle, while heart embryos were excised by making incision of the seeds with tweezers. For later-stage embryos, from torpedo to mature green, embryos were carefully extracted by puncturing the seed coat and endosperm with tweezers, followed by pushing embryos out. Each released embryo was manually collected using microcapillaries, one by one. All procedures used in this study, including pollination for embryo preparation, sampling of the embryos, genomic DNA extraction, bisulfite library construction, and the processing of whole-genome bisulfite sequencing (WGBS) data, were previously described in detail (Yoo et al., 2021).

### Global DNA methylation analysis

Embryo DNA methylation data was divided into 50 bp fractional windows that contained at least three cytosines with at least five aligned reads in all stages for each cytosine context. For the circular global map, we used 5kb windows that contained more than ten cytosines with at least five reads for each methylation context.

### Small RNA-Seq analysis

The small RNA-Seq dataset was obtained from the NCBI GEO (GSE98553, GSE132066, GSE152971) (Lutzmayer et al., 2017; Plotnikova et al., 2019; Papareddy et al., 2020). We performed adapter trimming, quality control, and 18-to-26 nt-length read selection using Trimmomatic (0.39) and Cutadapt (v3.4). The pre-processed reads were mapped to rRNA/tRNA/snRNA sequences (RNACentral, v17) using bowtie (v1.3.0, with parameters -v 1 -m 0 -a) to filter out structural non-coding RNA reads. The remaining reads were first aligned using bowtie (1.3.0, -v 0 -m 0 -a), and then the aligned reads were re-aligned with ShortStack (v3.8.5, with parameters –align_only –mismatches 0 –mmap u –bowtie_m 1000 –ranmax 3) using the TAIR10 genome sequence as a reference. Reads aligned to known microRNA precursors were removed using Bedtools intersect (v2.30.0). The microRNA-free reads were then used as input for downstream analysis. Small RNA read clustering was conducted in a sample-by-sample manner using ShortStack (with parameters –dicermin 20 –dicermax 24 –foldsize 300 –pad 1(or 75), –mincov 1.0rpmm –nohp) (Johnson et al., 2016).

### Average length of merged windows for the methylated block

To calculate the average length of the regions where consecutive methylated probes were present at each stage of embryo development, we used 10 bp windows with at least one methylation site and at least five aligned reads. We merged windows with methylation levels above 5%, 10%, or 15% in all stages for each cytosine context if they were closer than 40 bp apart. The resulting merged windows were used to determine the average length of the methylated regions at each stage.

### Relationship between sRNA clustering and spreading of DNA methylation

To identify regions with consecutive CHH methylation and sRNA clustering simultaneously in all stages of embryo development, we selected regions with consecutive CHH methylated probes in at least three out of five stages and a methylation level cutoff of 15%, along with sRNA clusters common in at least five out of eight stages. We divided the consecutive CHH methylated regions into two groups based on whether they overlapped with sRNA clustering or not. The average length of the regions with or without sRNA clustering was then calculated.

### Classification of TE by CMT2 or RdDM dependency

To determine the dependency of TE methylation on CMT2 and RdDM pathways, we utilized previously identified lists of differentially methylated regions (DMRs) (Stroud et al., 2013), and recently derived CHH methylation pathway DMR classifications (Hsieh et al., 2023): DMRs that are regulated by the CMT2 or RdDM pathways in both leaf and embryos are classified as cCMT2 DMRs or cRdDM DMRs, respectively. DMRs that are regulated by CMT2 or unmethylated in the leaf and become newly targeted by the RdDM pathway in embryo are classified as eRdDM DMRs. Here, TEs were allocated into the three DMR classes using bedtools intersect -wa -a TE list -b DMR list. TEs allocated to more than one DMR class were removed, and only TEs associated with a single type of DMR class chosen for further analyses.

## Results

### DNA methylation dynamics during *Arabidopsis* embryo development

**Figure 1:**
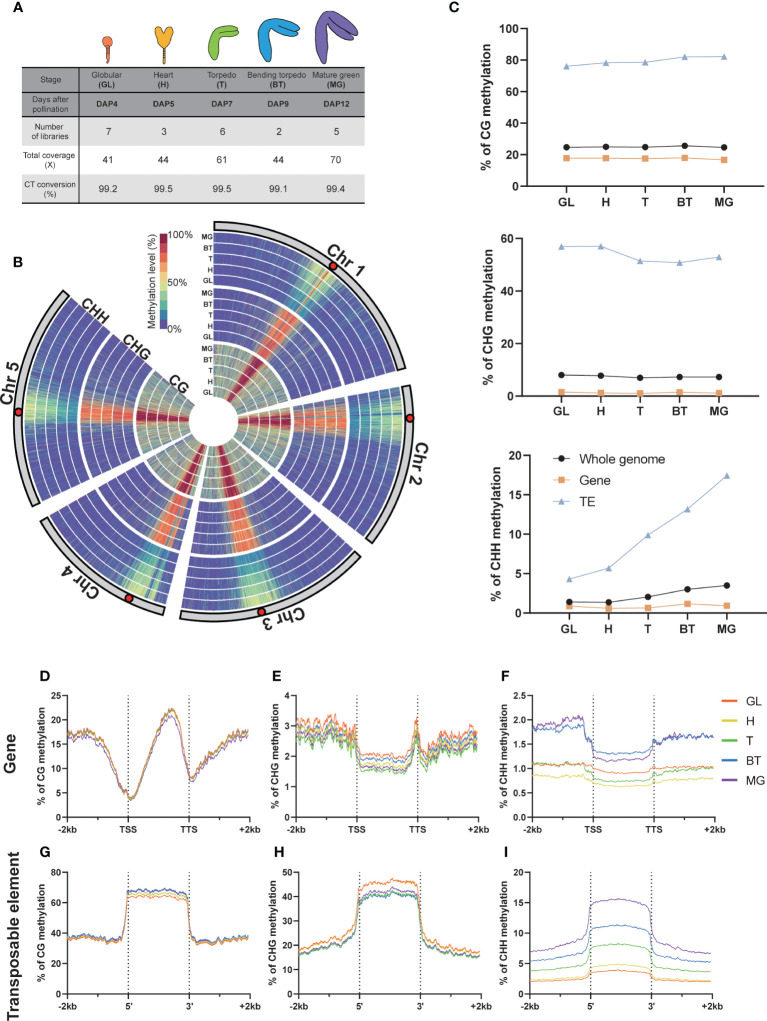
DNA methylation in global and genomic features. **(A)** Whole Genome Bisulfite Sequencing (WGBS) libraries used in this study. The percentage of C-to-T conversion was calculated by measuring 100% minus the methylation level of plastids that are known to be unmethylated. DAP, days after pollination. **(B)** Circular heatmap displaying the global DNA methylation levels during embryo development, with the inner circle representing the earlier developmental stages. GL, globular; H, heart; T, torpedo; BT, bending torpedo; MG, mature green. **(C)** The average DNA methylation levels by the different genomic features. **(D-I)** The DNA methylation levels of genomic features and their surrounding regions are shown.

We utilized our recently reported optimized method to profile the genome-wide *Arabidopsis* embryo methylome at five developmental stages: DAP4, 5, 7, 9, and 12, which correspond to the globular, heart, torpedo, bending torpedo, and mature green stage embryos, respectively (**Figure 1A**) (Yoo et al., 2021). All libraries were made up of at least two biological replicates with robust CT conversion (>99%) and genome coverage (>40X), complying with the recommended standard for methylation data analysis (Ziller et al., 2015) (**Figure 1B**).

As previously reported (Cokus et al., 2008; Lister et al., 2008), *Arabidopsis* chromosomes exhibited high levels of pericentromeric methylation in all cytosine contexts, due to the accumulation of repetitive sequences and heterochromatic TEs (**Figure 1B**). Consistent with previous reports (Bouyer et al., 2017; Kawakatsu et al., 2017; Lin et al., 2017; Papareddy et al., 2020), CG methylation levels remained relatively constant throughout embryo development, whereas non-CG methylation was more dynamic (**Figure 1C**). In particular, as others have reported, there was a gradual increase in global CHH methylation levels in pericentromeric regions during embryo development (**Figure 1B**), which culminated as the seeds mature (**Figure 1C**). The increase in CHH methylation levels primarily resulted from increases at TEs, whereas gene body methylation levels in all cytosine contexts remained relatively stable (**Figure 1C**). CHH methylation levels in gene bodies increased only slightly (**Figure 1C**). CHG methylation levels in TEs showed a slight decrease, particularly from DAP7 to DAP9 (**Figure 1C**), but otherwise remained relatively stable as the seeds mature.

We next analyzed the methylation dynamics of *Arabidopsis* embryo development within genomic features, focusing on genes and TEs (**Figure 1**). As observed genome-wide, we found that the methylation change from the beginning to end of embryogenesis was small in genic regions in all contexts (<0.7%; **Figures 1D–F**). However, we observed distinct gene body methylation levels for both CHG and CHH contexts at each developmental stage (**Figures 1E, F**), indicating a coordinated change in methylome status during development. For CHH methylation at the 5’ and 3’ ends of genes, there was a slightly larger increase (1%) that was distinguishable from the globular-torpedo to the bending torpedo-mature green stages, likely due to the presence of overlapping TE sequences in the flanking regions of genes (**Figure 1F**). As noted above, the torpedo embryo stage at DAP7 showed the lowest CHG methylation levels (**Figures 1C–E**).

At TEs, CG methylation increased marginally throughout embryogenesis (**Figures 1G–I**). CHH methylation levels at TEs, as previously noted, demonstrated a profound and continuous increase during development (**Figure 1I**). Interestingly, a striking increase was shown in CHH methylation at TEs from DAP5 to DAP7, which coincided with a decrease in CHG methylation levels (**Figures 1C–H**). This observation may indicate that CHG and CHH methylation work together in a coordinated manner to repress TEs during heart (H) to torpedo (T) transition phase, which marks the beginning of maturation after pattern formation.

### CHH methylation spreading is accompanied by sRNA cluster expansion during embryo development

**Figure 2:**
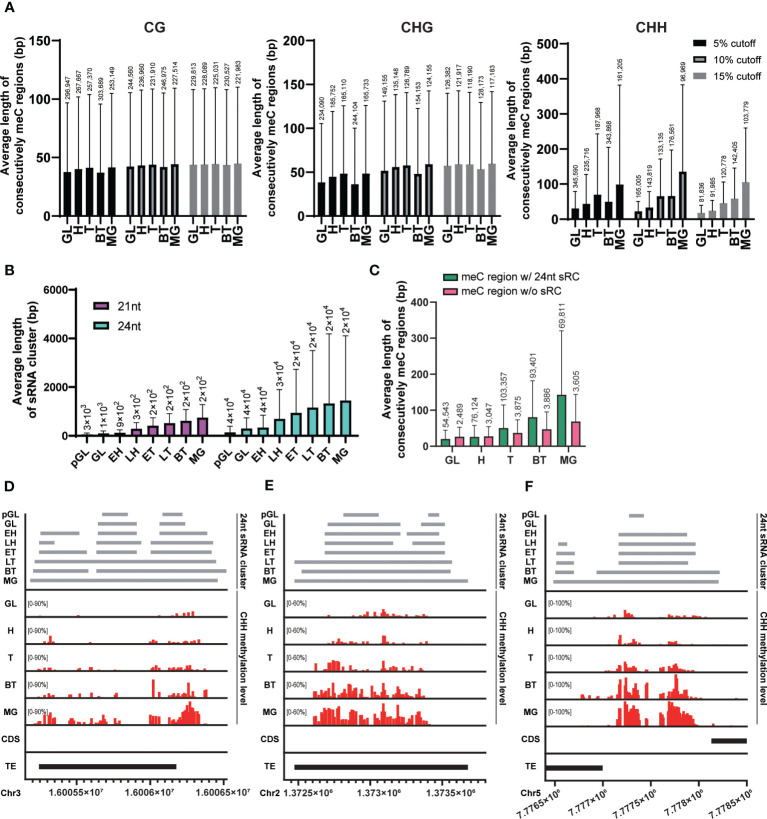
Spreading of CHH methylation and expansion of sRNA expression. **(A)** The average length of regions where the methylated probes exist consecutively in each cytosine context during embryo developmental stage. **(B)** The average length of merged sRNA clusters in each embryo developmental stage. **(C)** The average length of regions where consecutively CHH-methylated overlapped or did not overlap with merged 21nt or 24nt sRNA clusters (sRC) during each embryo developmental stage. **(A-C)** The height of box and whisker indicate mean and standard deviation, respectively. The number of loci (for the methylated regions and sRNA clusters) are indicated on each whisker. **(D-F)** Example regions illustrating the spread of CHH methylation. GL, globular; H, heart; T, torpedo; BT, bending torpedo; MG, mature green.

In mammalian embryogenesis, a unique form of *de novo* CG methylation occurs through the spreading of methylation from cis-regulatory regions to distal CpG sites (Turker, 2002). To investigate whether this phenomenon of methylation spreading also occurs in plants during embryo development, we calculated the average length of the region where CHH methylated probes exist consecutively at each embryo developmental stage. We merged windows with methylation levels above 5%, 10%, or 15% in all stages for each cytosine context if they were closer than 40 bp apart. As the embryos developed, the average length of consecutively methylated regions gradually increased, especially when we used higher methylation cutoffs (15%; **Figure 2A**). This result suggests that genome-wide CHH methylation spreads as the embryo matures.

The RdDM pathway plays a critical role in *de novo* methylation in *Arabidopsis* (Matzke and Mosher, 2014; Chow et al., 2020; Erdmann and Picard, 2020), however, it is possible that sRNAs are only required for the initial CHH methylation setting during early embryogenesis. This initial CHH methylation could then act as a signal for its spreading to nearby sites at later stages of embryogenesis, similarly to CG methylation in mammals. In this scenario, CHH methylation spreading would occur even in the absence of matching sRNAs to nearby sites. Alternatively, sRNA clusters corresponding to neighboring regions may be continuously required for the spreading of CHH methylation. We measured the average length of 24nt sRNA clusters that are merged when they exist within 75 bp at each stage using publicly available sRNA expression data during embryogenesis (Papareddy et al., 2020). As the embryos developed, the average length of merged sRNA clusters for 24nt sRNAs gradually increased (**Figure 2B**). This suggests that expression of sRNAs corresponding to nearby sites is still required for the methylation of neighboring unmethylated CHH sites during embryo development.

To investigate the relationship between the expansion of sRNA expression and the spreading of CHH methylation during embryo development, we compared the average length of regions where consecutive CHH-methylated windows overlapped with merged 24nt sRNA clusters. Our results revealed a linear increase in the length of the sRNA clusters during development in CHH overlapping regions, demonstrating that there is a positive correlation between the spreading of CHH methylation and expansion of sRNA-producing regions (**Figure 2C**, green bars). CHH methylated regions were also expanded in the absence of sRNA clusters as embryos matured (**Figure 2C**, pink bars). However, these regions were formed of far fewer loci and reflected less extensive CHH spreading. In addition, when we mapped the position of these non-sRNA overlapping CHH methylated regions, we found that they were tightly centromeric, and as such likely to be conferred by CMT2 (**Supplementary Figure S1**). To further investigate the positive correlation of CHH methylation spreading with sRNA clusters, we visualized individual overlapping loci, and observed that expanding sRNA expression was coincident with CHH methylation spreading and accumulation (**Figures 2D–F**), providing support for previous observations that increased embryo CHH methylation is conferred by the RdDM pathway (Hsieh et al., 2023).

Taken together, our data suggest that the RdDM pathway plays an important role in both initiating and spreading CHH methylation to nearby regions, as well as in the accumulation of CHH methylation levels. This can be achieved, at least in part, by the continuous expression of sRNA clusters followed by expression of sRNA clusters in neighboring regions, resulting in wider CHH methylation regions and higher CHH methylation levels at later stages of embryogenesis.

### Chromosomal location shapes distinctive methylation dynamics in CMT2 and RdDM DMRs

CHH methylation is known to be regulated by two pathways; the RNA-independent CMT2 pathway and the RdDM pathway (Zemach et al., 2013; Matzke and Mosher, 2014; Kenchanmane Raju et al., 2019). RdDM targets are methylated by *de novo* methyltransferases, DRM1 and 2. Recent papers described the delineation of embryo CHH loci into classes based on their mechanism of catalysis using *drm1/2* and *cmt2* mutants (Kawakatsu et al., 2017; Hsieh et al., 2023). Briefly, if an embryonic CHH DMR is targeted by CMT2 both in the vegetative (leaves) and reproductive embryo stages, that DMR is denoted as a conserved CMT2 (cCMT2) DMR. If a DMR is targeted by DRM1/2 both in leaves and embryos, that DMR is denoted as a conserved RdDM (cRdDM) DMR. If a DMR is targeted by CMT2 or unmethylated in leaves but becomes exclusively targeted by DRM1/2 in embryos, that DMR is denoted an embryonic RdDM (eRdDM) DMR (Hsieh et al., 2023)

**Figure 3:**
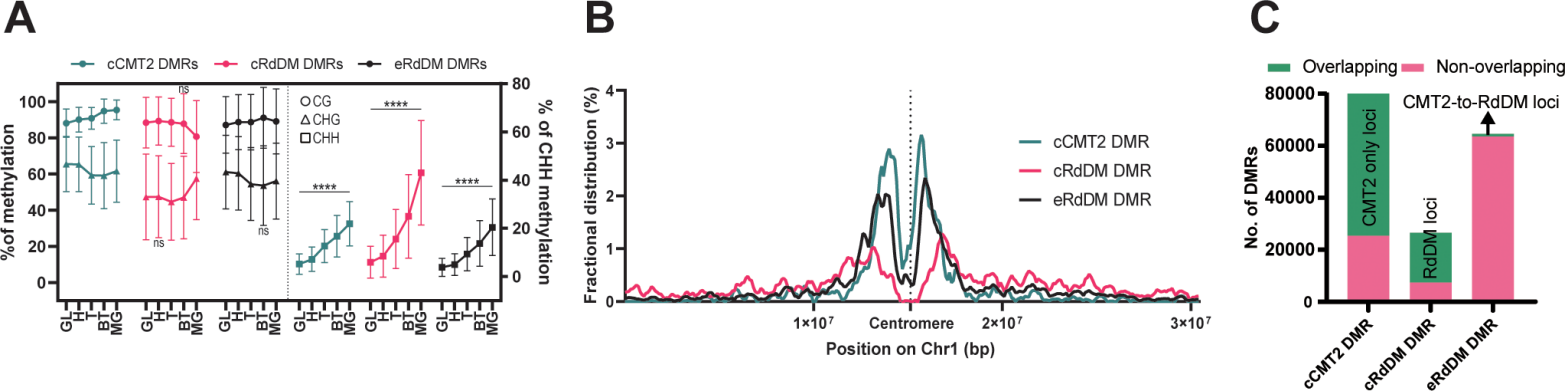
Characteristics of three types of DMRs. **(A)** The average DNA methylation levels of the three types of DMRs were measured at each stage. The levels of mCG and mCHG are shown on the left side, while the levels of mCHH were shown on the right side. The error bar represents the standard deviation. The p-value for each developmental stage was determined by comparing it to the prior stage. CHH methylation levels during embryo development were significantly changed in all stages (p < 0.0001). Similarly, the p-values for mCG and mCHG also showed significant changes, except for the stages denoted as ‘ns’ (not significant). **(B)** Fractional distributions of the position of the three types of DMRs on chromosome1 were shown. **(C)** The three DMR classes used in this study were compared with the previously identified DMRs using a similar approach for the regions found in the seedling stage (He et al., 2021). The counts of DMRs, defined at the loci from (He et al., 2021), were colored in green, and those not found were colored in pink.

In order to understand the methylation dynamics of these three DMR classes during development, we divided our data according to DMR class and examined their average methylation level during embryogenesis (**Figure 3A**, also see Methods). Average CG methylation levels of cCMT2 DMRs increased from DAP4 to DAP12 (*p* < 0.0001), whereas those of cRdDM DMRs decreased during the same period (*p* < 0.0001) (**Figure 3A**). Both the levels and pattern of CG methylation in eRdDM DMRs laid between those of cCMT2 DMRs and cRdDM DMRs (**Figure 3A**). Average CHG methylation level change was unique, whereby cCMT2 and eRdDM DMRs showed decreases from DAP5 to DAP7 (p< 0.0001 for cCMT2 DMRs and p < 0.0001 for eRdDM DMRs) whereas cRdDM DMRs showed large increases in CHG methylation from DAP7 to DAP12 (p<0.0001) (**Figure 3A**). CHH methylation levels were continuously increased during embryogenesis in all classes (**Figure 3A**). The most dynamic epigenetic change observed during embryogenesis remained the constant increase in CHH methylation, and among them, cRdDM-targets displayed the largest change. This was particularly striking from DAP9 to DAP12, reaching 60% CHH methylation levels at DAP12 (**Figure 3A**).

To explore the overall location of CMT2/RdDM-dependent DMRs, we mapped cCMT2-, cRdDM-, and eRdDM DMRs to chromosomes. All three classes were enriched in pericentric chromatin (**Figure 3B**). However, consistent with previous reports, cCMT2 DMRs were almost entirely located in pericentromeric regions (He et al., 2021) (**Figure 3B**, green trace), whereas cRdDM DMRs were much more evenly spread throughout chromosome arms (**Figure 3B**, pink trace). Interestingly, eRdDM DMRs were most similar in profile to cCMT2 DMRs, with clear pericentromeric enrichment, although they demonstrated comparative increases in chromosome arm enrichment (**Figure 3B**, black trace). These data indicate that chromosomal location, which is highly associated with local chromatin state, may be key to determining CHH enzyme dependency, consistent with previous reports (Sigman and Slotkin, 2016).

Recently, it was reported that some regions regulated by CMT2 in wild-type *Arabidopsis* seedlings are accessed by the RdDM pathway in the absence of the DDM1 nucleosome remodeling factor, which in wild-type plants mediates heterochromatic DNA methylation in all cytosine contexts (Zemach et al., 2013; He et al., 2021). These regions were referred to as “CMT2-to-RdDM loci” by the authors. Some eRdDM DMRs exhibited similar traits to CMT2-to-RdDM loci, as they were initially methylated by CMT2 in somatic tissues and subsequently switch to the RdDM pathway after fertilization during embryonic development (Hsieh et al., 2023). This prompted us to investigate the extent of overlap between eRdDM DMRs and CMT2-to-RdDM loci by He et al. We conducted a parallel comparison of these regions and found that most of the DMRs were not shared between two datasets (**Figure 3C**), indicating that eRdDM DMRs are distinct from the CMT2-to-RdDM loci.

### Methylation levels of TE- flanking regions differ according to CHH regulatory mechanisms

**Figure 4:**
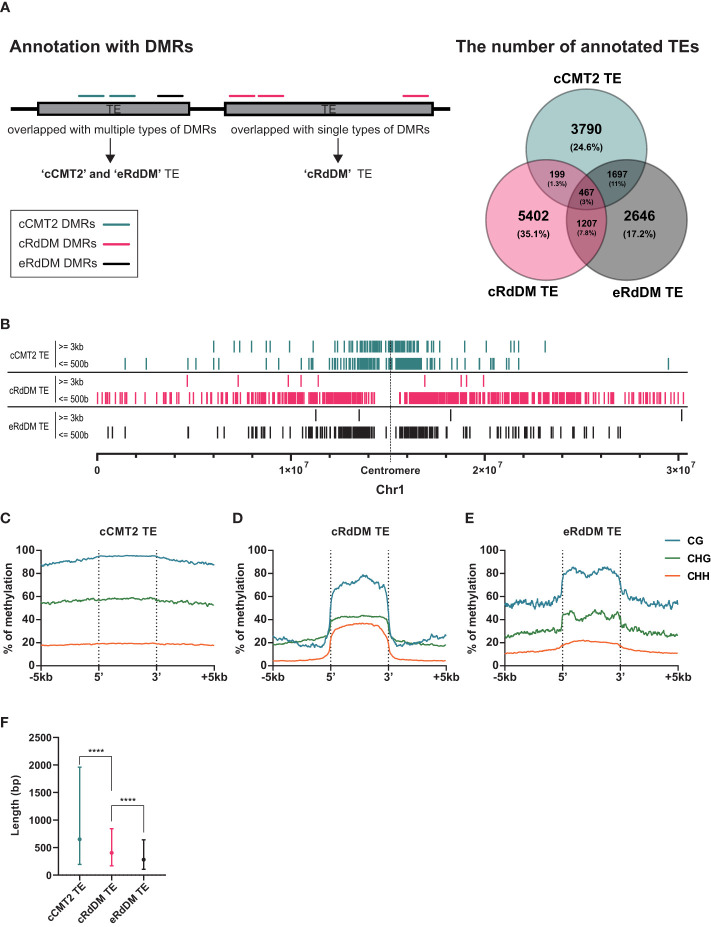
Distinctive characteristics of TEs categorized into three types. **(A)** A diagram explaining how to categorize TEs is shown on the left side, and the classified TEs are compared on the right side. Only the exclusive parts were used for the following analyses. **(B)** The positions of classified TEs divided by length on chromosome 1 were analyzed. **(C-E)** DNA methylation levels of the three classified TEs and their surrounding regions were analyzed. **(F)** The length of the three classified TEs was compared. Dots and error bars indicate the average and standard deviation, respectively. **** indicates a significant difference (*p < 0.0001*).

The purpose of CHH methylation in *Arabidopsis* is widely accepted to be TE silencing, to prevent insertional mutagenesis in key tissues, such as the developing embryo. To explore TE dynamics during embryo development, we categorized TEs based on their dependency on CHH methylation pathway. As for our whole-genome analyses, we annotated TEs according to the three DMR classes outlined above (cCMT2, cRdDM and eRdDM) (Hsieh et al., 2023), and calculated the proportion of each (**Figure 4A**). Then, only those TEs annotated exclusively with a single category of DMR were used for further analyses; 3,790 for cCMT2 TEs, 5,402 for cRdDM TEs and 2,646 for eRdDM TEs (**Figure 4A**).

To investigate the overall location of TEs depending on their CHH mechanistic class, we mapped cCMT2, cRdDM, and eRdDM TEs to the chromosomes. Similar to the chromosomal location of each CHH DMR (**Figure 3B**), we found cCMT2 TEs were more highly concentrated in pericentromeric regions than other classes of TEs. Furthermore, we observed that longer cCMT2 TEs (≥ 3kb) were more highly concentrated in pericentromeric regions compared to shorter cCMT2 TEs (≤ 500 bp) (**Figure 4B**). In contrast, cRdDM TEs were located throughout the chromosomes and most of them were shorter than 500bp (**Figure 4B**). Interestingly, the majority of eRdDM TEs were also shorter than 500bp, similar to cRdDM TEs (**Figure 4B**), indicating that accessible length is a fundamental attribute of loci targeted by RdDM. Additionally, we observed that eRdDM TEs were predominantly located similarly to the shorter cCMT2 TEs, with a slight shift towards chromosomal arms (**Figure 4B**).

We compared the overall cytosine methylation levels for the three TE classes, mainly revealing differences in flanking regions. Consecutive high levels of methylation in all contexts surrounding TE bodies was observed in cCMT2 TEs (**Figure 4C**), but not in cRdDM TEs (**Figure 4D**). This is consistent with previous observations of comprehensive heterochromatin formation and DNA methylation in pericentromeric regions, since cCMT2 TEs were almost all pericentromeric, and cRdDM TEs were much more spread along the chromosome (**Figure 4B**) (Zemach et al., 2013; Yelagandula et al., 2014; Zhao et al., 2019; Bourguet et al., 2021). eRdDM TE methylation levels were intermediate between the other two classes, displaying the same clear delineation of CG and CHG methylation levels between TE bodies and flanking regions as cRdDM sites, but much less so for CHH methylation (**Figure 4E**).

We next examined the average length of TEs classified into the three DMR classes. cCMT2 TEs tended to be longer than cRdDM TEs, although with large variation (**Figure 4F**, *p* < 0.0001). eRdDM TEs were even shorter in size than cRdDM TEs (**Figure 4F**, *p* < 0.0001). Considering that 29% of eRdDM TEs were cCMT2 TEs in leaves (**Figure 4A**, **Supplementary Figure S2**), this is quite intriguing. Based on their size and location, this result raises the idea that eRdDM TEs that originated as CMT2 TEs in leaves were shorter, less-heterochromatic CMT2 sites. We speculate that these sites transitioned to RdDM targets due to changes in local chromatin organization and the presence of highly robust RdDM activity during the developmental transition upon fertilization.

### TE size is a critical factor for CHH dependency even within the same TE superfamily

To test whether the composition of TE superfamilies within each TE class is correlated to CHH-conferring enzyme dependency, we investigated the proportion of representative TE superfamilies within each TE class. In *Arabidopsis*, retroelements represent the most common TE type, comprising ~10Mb of the genome, closely followed by DNA transposon orders Helitrons (8 Mb) and TIR elements (7 Mb) (Ahmed et al., 2011). TIR elements are so called due to their Terminal Inverted Repeats, and two of the most frequent TIR superfamilies in *Arabidopsis* are Mutator-like (MuDR) which is also one of the most mobile elements (Quadrana et al., 2016), and En-Spm. Retroelements can be further delineated into LTR (long terminal repeat) TEs such as Gypsy and Copia [another highly mobile elements, (Quadrana et al., 2016)], and non-LTRs such as LINEs and SINEs (Quesneville, 2020).

**Figure 5:**
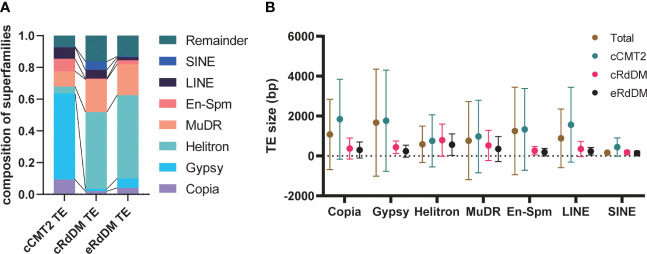
Characteristics of the three TE classes. **(A)** The composition of the seven superfamilies in three TE classes were analyzed. Remainder indicates the TEs that are not included in the seven superfamilies. **(B)** The average TE size according to superfamilies was analyzed. Dots and error bars indicate the average and standard deviation, respectively.

We examined whether any TE superfamilies were enriched in a particular TE class. As shown in **Figures 5A, B**, cCMT2 TEs were highly enriched for Gypsy LTR retrotransposons, which is known to be the longest TE in mean length and concentrated in pericentromeric regions (Quesneville, 2020). So most Gypsy elements acquire CHH methylation *via* the CMT2 pathway. In contrast, both cRdDM and eRdDM TEs were depleted for LTR targets, instead enriched for the Helitron and MuDR superfamilies of TEs, of which the mean lengths were significantly shorter than LTR retrotransposons (**Figures 5A, B**). All TE superfamilies, regardless of their average length, exhibited the same trend: longer TEs were targeted by the CMT2 pathway, while shorter TEs were targeted by the RdDM pathway, within each family (**Figure 5B**). For instance, SINEs were generally the shortest TE among the seven different superfamilies we examined. However, there were SINE TEs targeted by CMT2 which were, although long relative to other SINEs, were still shorter than RdDM-targeted TEs in the Helitron superfamily (**Figure 5B**). In addition, the mean length of eRdDM TEs were shorter than cRdDM TEs even within each superfamily (**Figure 5B**). This suggests a sequence based mechanism influences CHH methylation targeting, whereby once an element reaches a certain size, the sequence composition is such that CHH targeting switches from RdDM to CMT2 based mechanisms. Shorter TEs that are unmethylated, or even CMT2-regulated in leaves, may become accessible to the RdDM pathway after the transition to reproductive development in the embryo. Taken together, and consistent with a previous report (Zemach et al., 2013), our data highlight that TE size, relative to the superfamily, is one of the most important factors for CHH methylation targeting mechanisms.

All three DMR classes of CHH-methylated loci in embryo were found across all TE superfamilies (**Figures 5A, B**). Next we examined methylation dynamics of each TE family during embryo development. In general, cCMT2 TEs showed uniform methylome dynamics throughout the superfamilies (**Supplementary Figure S3A**), whereas both cRdDM- and eRdDM TEs showed more variable methylome dynamics in each superfamily (**Supplementary Figures S3B, C**). Consistent with the result shown in **Figure 3A**, while all the TEs showed gradual increase of CHH methylation, cRdDM TEs showed the most striking increase in CHH methylation for all superfamilies (**Supplementary Figure S3B**, cRdDM_CHH). CG methylation levels were decreased specifically at DAP12 (**Figure 3A**), an all seven superfamilies of cRdDM TEs followed a similar pattern (**Supplementary Figure S3B**, cRdDM_CG). It is tempting to speculate that the decrease in CG methylation in cRdDM TEs located in chromosomal arms might induce an active demethylation mechanism, resulting in an increase in sRNA generation and CHH *de novo* methylation. The large increase in CHH methylation of TEs during late embryo development is supported by previous reports that genes involved in RdDM such as AGO4, DRM2 and DMS3, and concomitant sRNAs, showed highest expression during late embryogenesis (Belmonte et al, 2013; Bouyer et al., 2017; Papareddy et al., 2020). Interestingly, Helitron transposons, which were the most enriched in terms of copy number and genome coverage among the superfamilies we analyzed (**Figure 5A**), showed the lowest CG and CHG methylation levels in RdDM at all stages (**Supplementary Figures S3B, C**). Taken together, our data suggest that the CHH regulatory pathways mediating TE methylation dynamics are independent of TE superfamily.

### The length of each TE class can impact methylation levels during embryogenesis

**Figure 6:**
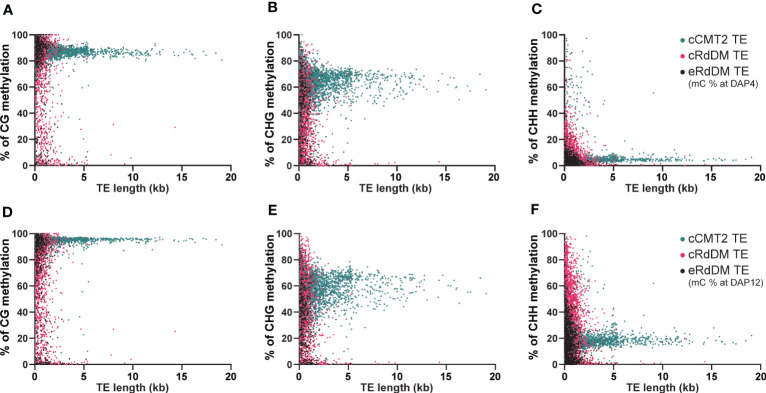
Analysis of DNA methylation levels according to the length of TEs. The DNA methylation levels at globular embryo (DAP4) **(A-C)** and those of mature green embryo (DAP12) **(D-F)** were used for this analysis. The DNA methylation levels on TEs were measured by calculating the mean percentage of mC at every cytosine site.

We next investigated the impact of TE length on embryo methylation levels and dynamics in different CHH regulatory pathways at DAP4 (**Figures 6A–C**) and DAP12 (**Figures 6D–F**). Almost all longer (>3 kb) TEs belonged to the cCMT2 TE category in all cytosine contexts (green in **Figures 6A–F**), which was consistent with the findings in **Figures 4F**, **5B**. Moreover, methylation levels for cCMT2 TEs tended to converge at a specific level as TE length increased in all cytosine contexts. Methylation levels increased from DAP4 to DAP12, especially in CG and CHH contexts (**Figures 6A–F**). This suggests that cCMT2 TEs maintain specific ranges of methylation levels regardless of their length, consistent with their collective methylation pattern around the centromere, observed above (**Figure 4C**; **Supplementary Figure S3A**). The CG methylation range for cCMT2 TEs was broader at DAP4, becoming narrower at DAP12 (**Figures 6A–D**). Interestingly, the opposite trend was observed for CHH methylation, as DAP12 exhibited a broader range of CHH methylation. These results indicate that as the embryo matures, cCMT2 TEs gain a higher and more uniform level of CG methylation (around 95%) whereas CHH increases are more variable, resulting in a wider range of levels DAP12. Intriguingly, although many cCMT2 TEs displayed low CHH methylation at DAP4 (around 5%), some short cCMT2 TEs exhibited very high CHH methylation (**Figure 6C**).

Unlike cCMT2 TEs, the majority of both RdDM TEs were found to reside in shorter TEs, with eRdDM TEs being slightly shorter than cRdDM TEs (**Figures 6A–F**). The overall methylation patterns, particularly for CG and CHG methylation, were similar between cRdDM TEs and eRdDM TEs (**Figures 6A–E**). The eRdDM TEs exhibiting high CG methylation levels showed almost the same levels as those in cCMT2 TEs, suggesting that these eRdDM TEs in the reproduction stage likely originated from cCMT2 TEs in the vegetative stage. CHH methylation levels of eRdDM TEs were found to be lower than those in cRdDM TEs at DAP4, and the increasing rate of CHH methylation of during embryogenesis was slower. Therefore, the highest CHH methylation level observed in eRdDM TEs was around 45%, compared with cRdDM TEs, which exhibited levels around 95% at DAP12 (**Figures 6C–F**).

### Cytosine frequency and methylation levels in different TE classes

**Figure 7:**
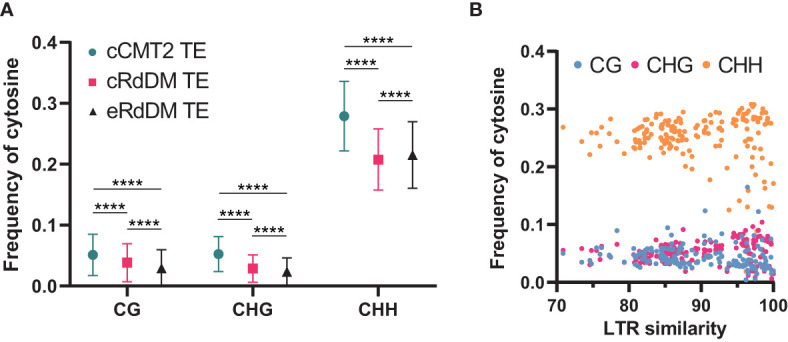
Calculation of methylation context frequencies on TEs. **(A)** The frequencies of each methylation context on TEs were calculated by dividing the number of the same methylation context with the length of TE. **** indicates a significant difference (*p < 0.0001*). **(B)** The frequencies of each cytosine context on *de novo* intact LTRs were analyzed. Intact LTRs and their similarity were defined using a previous report (Wang and Baulcombe, 2020).

While it has been known that heterochromatic TEs have higher GC content than euchromatic TEs (Zemach et al., 2013), examining the frequency of all cytosine contexts may provide insights into the relationship between CHH regulatory dependency of TEs and their sequence composition. Our analysis revealed that cCMT2 TEs exhibited significantly higher cytosine frequency across all contexts compared to cRdDM TEs and eRdDM TEs (**Figure 7A**. p < 0.0001). eRdDM TEs demonstrated the lowest CG and CHG frequency, but slightly higher CHH frequency compared to cRdDM TEs (**Figure 7A**). We measured the frequencies of CG, CHG and CHH in the three TE classes as well as those of all Arabidopsis TEs as a reference based on each TE superfamily. We also calculated the expected frequencies using arithmetic calculations (**Table 1**). As a result, we observed that the cytosine frequencies of most cCMT2 TEs were higher than two RdDM classes, as well as all TEs across all cytosine contexts. However, the patterns of cytosine frequencies differed between cRdDM TEs and eRdDM TEs, depending on the context. Both cRdDM TEs and eRdDM TEs exhibited lower CHG frequency compared to all TEs except for the SINE superfamily in cRdDM TEs (0.052) (**Table 1**; **Figure 7A**). Between cRdDM TEs and eRdDM TEs, eRdDM TEs showed lower CHG frequency but higher CHH frequency, except for the En-Spm superfamily (**Table 1**, **Figure 7A**). Notably, the CHH frequency of cRdDM TEs was the lowest among the three TE classes, suggesting that TEs with low CHH frequency are preferentially targeted by the RdDM pathway during vegetative growth. Since the region with the highest CHG frequency in cCMT2 TEs is likely to be more heterochromatic than the CHG region in RdDM TEs, our analysis suggests that the difference between cCMT2 TEs and eRdDM TEs may be attributed, at least in part, to the higher CHG frequency observed in cCMT2 TEs. This further implies that shorter CMT2 TEs with lower CHG frequency may contribute to epigenetic reprogramming during the transition from the vegetative to the reproductive lifecycle, and they may be newly targeted by the RdDM pathway, becoming eRdDM TEs.

**Table 1:** The frequencies of cytosine methylation contexts on TE superfamily.

Class		All TEs			cCMT2 TEs			cRdDM TEs			eRdDM TEs		
mC context		CG	CHG	CHH	CG	CHG	CHH	CG	CHG	CHH	CG	CHG	CHH
**Expectation**		**0.063**	**0.047**	**0.28**	**0.063**	**0.047**	**0.28**	**0.063**	**0.047**	**0.28**	**0.063**	**0.047**	**0.28**
**Observation**	**Copia**	**0.039**	**0.042**	**0.261**	**0.042**	**0.052**	**0.274**	**0.038**	**0.031**	**0.250**	**0.032**	**0.027**	**0.259**
	**Gypsy**	**0.055**	**0.053**	**0.280**	**0.057**	**0.055**	**0.290**	**0.049**	**0.037**	**0.257**	**0.035**	**0.032**	**0.270**
	**Helitron**	**0.027**	**0.023**	**0.201**	**0.034**	**0.040**	**0.240**	**0.030**	**0.022**	**0.195**	**0.024**	**0.020**	**0.205**
	**MuDR**	**0.036**	**0.030**	**0.220**	**0.049**	**0.050**	**0.265**	**0.036**	**0.026**	**0.213**	**0.032**	**0.025**	**0.313**
	**En-Spm**	**0.050**	**0.045**	**0.252**	**0.049**	**0.052**	**0.260**	**0.063**	**0.025**	**0.236**	**0.053**	**0.031**	**0.222**
	**LINE**	**0.054**	**0.048**	**0.258**	**0.050**	**0.060**	**0.291**	**0.061**	**0.042**	**0.231**	**0.049**	**0.040**	**0.259**
	**SINE**	**0.059**	**0.048**	**0.245**	**0.071**	**0.055**	**0.289**	**0.070**	**0.052**	**0.251**	**0.035**	**0.034**	**0.269**

Note: Observed frequency was calculated by dividing each the counts of cytosine context by the length of TE. The background color was determined by higher (green) or lower (orange) than the frequency of all TEs (blue). Expected frequencies of CG, CHG and CHH were calculated as following formula; CG (0.25x0.25), CHG (0.25x0.75x0.25), CHH (0.25x0.75x0.75x2, double strands).

Recent research has shown that although euchromatic TEs are usually regulated by RdDM, young and LTR transposons in euchromatin are typically regulated by CMTs. Furthermore, longer and younger LTR transposons tend to have higher CHG methylation levels (Wang and Baulcombe, 2020). We analyzed the cytosine frequencies of predicted *de novo* LTRs using publicly available data (Wang and Baulcombe, 2020) and found that intact LTRs with higher LTR sequence similarity, which are thought to be more recently introduced in the genome, displayed diverse cytosine frequencies. By contrast, older LTRs with low similarity due to mutations had similar cytosine frequencies, suggesting they have decayed over time during evolution (**Figure 7B**). Taken together, our findings highlight the correlation between length, location, cytosine ratio, DNA methylation, and regulatory pathways of TEs.

## Discussion

In embryos, a programmed and gradual increase in CHH methylation throughout seed development has been reported in several dicot plants, including *Arabidopsis* (Bouyer et al., 2017; Kawakatsu et al., 2017; Lin et al., 2017), *Brassica rapa* (Grover et al., 2020), soybean (Lin et al., 2017), and chickpea (Rajkumar et al., 2020). However, the epigenetic pathways involved in this methylation reconfiguration have not been fully elucidated. In *Arabidopsis*, this gradual increase in CHH methylation is accompanied by at least two waves of massive sRNAs accumulation produced by thousands of TEs (Papareddy et al., 2020). Here, we provide results consistent with previous studies that during *Arabidopsis* embryo development, the level of CHH methylation gradually increases (Bouyer et al., 2017; Kawakatsu et al., 2017; Lin et al., 2017), whereas CG and CHG methylation remain high and constant. Our study reveals that the gradual increase in CHH methylation during embryo development is accompanied by the spreading of methylation in many loci. We showed that this process coincided with the expansion of sRNA expression, which was highly correlated with the expansion of CHH-methylated regions, as well as the increase in CHH methylation levels. This reconfiguration of CHH methylation was associated with sRNA-dependent features and sRNA-independent enzymes such as CMT2. Indeed, the expression of sRNAs and associated enzymes were positively correlated with the resetting of CHH methylation (Papareddy et al., 2020).

Our study showed that the dependency of individual loci on previously defined CHH methylation pathways (Hsieh et al., 2023) is likely dictated by chromosomal location, with cCMT2 DMRs predominantly concentrated at pericentromeric regions, cRdDM DMRs distributed throughout the chromosomes, and eRdDM DMRs located in-between. eRdDM DMRs are unique from He et al.’s CMT2-to-RdDM loci, which were identified in *ddm1* mutants. Firstly, most of the DMRs are not shared between the eRdDM class and He et al.’s CMT2-to-RdDM loci. Secondly, eRdDM TEs have an average length of less than 500 bp, while the average length of TEs overlapped with CMT2-to-RdDM DMRs are over 4kb (He et al., 2021). These results suggested that eRdDM TEs are prone to undergo a natural conversion from heterochromatic to euchromatic state during developmental transition, likely due to the reprogramming of CHH methylation during the transition from the vegetative to reproductive life cycle. Thus, while eRdDM DMRs are targeted by CMT2 in the leaf, they become accessible to the RdDM pathway following CHH reprograming upon fertilization. This may be due to the fact that eRdDM TEs tend to be shorter and located towards the chromosomal arms compared to cCMT2 targets. In contrast, CMT2-to-RdDM loci are heterochromatic regions maintained by DDM1 and are thought to be stably regulated by CMT2 in the wild-type state.

Our analyses of TEs regulated by the three different CHH pathways revealed significant differences in terms of their size, methylation levels, and chromosomal location. Longer TEs tend to be targeted by CMT2 whereas shorter TEs are targeted by the RdDM pathway. Interestingly, this trend remains consistent even within the same superfamily. Our results demonstrated that the size of TEs even within each TE superfamily is the crucial factor for their CHH enzyme dependency, regardless of their classification into different superfamilies. Therefore, the size of the TEs appears to be a more important factor than their family or origin. Compared with cRdDM TEs or eRdDM TEs, cCMT2 TEs tend to maintain specific ranges of methylation levels, especially for longer TEs. Intriguingly, shorter cCMT2 TEs show more variations in non-CG methylation levels at DAP12 compared to DAP4, which is the opposite of what is observed in CG methylation levels. The majority of both cRdDM TEs and eRdDM TEs are short in length and exhibit similar overall methylation patterns. When analyzing the frequency of cytosines in relation to TE length, we found cCMT2 TEs had a higher cytosine frequency at all contexts than both cRdDM TEs and eRdDM TEs. Specifically, a significant difference in CHG frequency depending on the regulatory pathway was observed in that CHG frequency in cCMT2 TE superfamilies tended to be higher than expected (green in CHG of **Table 1**) which was not observed in cRdDM TEs and eRdDM TEs superfamilies (**Table 1**). This raises the possibility that shorter CMT2 TEs with lower CHG frequency may be prone to undergoing epigenetic reprogramming during the transition and becoming accessible to RdDM machinery.

Taken together, our findings highlight the correlation between length, location, cytosine ratio, DNA methylation, and regulatory pathways of TEs. These factors all contribute to the evolutionary trajectory of TEs and by examining these characteristics, we can gain insights into the underlying mechanisms by which TEs evolved in host genomes.

## Data availability statement

The data presented in the study are deposited in the NCBI, accession number PRJNA944400 (https://www.ncbi.nlm.nih.gov/bioproject/PRJNA944400).

## Author contributions

JL, KP, and YC designed the research. JL, KP, and SL performed the experiments. JL, KP, SL, JF, P-HH, RF, T-FH, and YC analyzed the methylome data. SS and CS analyzed small RNA clusters. JL, SL, T-FH, and YC wrote the manuscript. All authors contributed to the article and approved the submitted version.
