# Supplementary figures and images for: Distinct regulatory pathways contribute to dynamic CHH methylation patterns in transposable elements throughout Arabidopsis embryogenesis
Source: Front Plant Sci. 2023 Jun 8;14:1204279. doi: 10.3389/fpls.2023.1204279 (PMC10285158; doi:10.3389/fpls.2023.1204279)

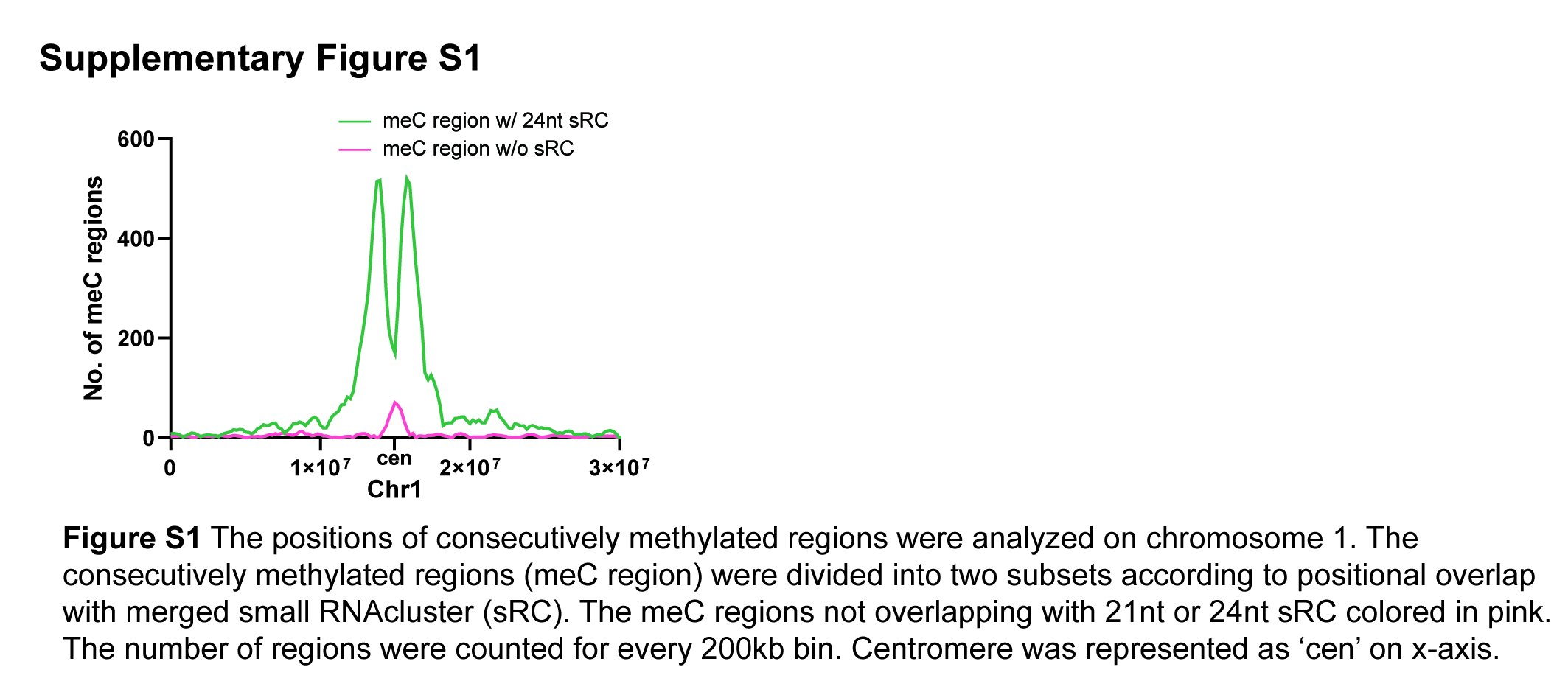

Supplement: Supplementary file 1 [file Image_1.tif]

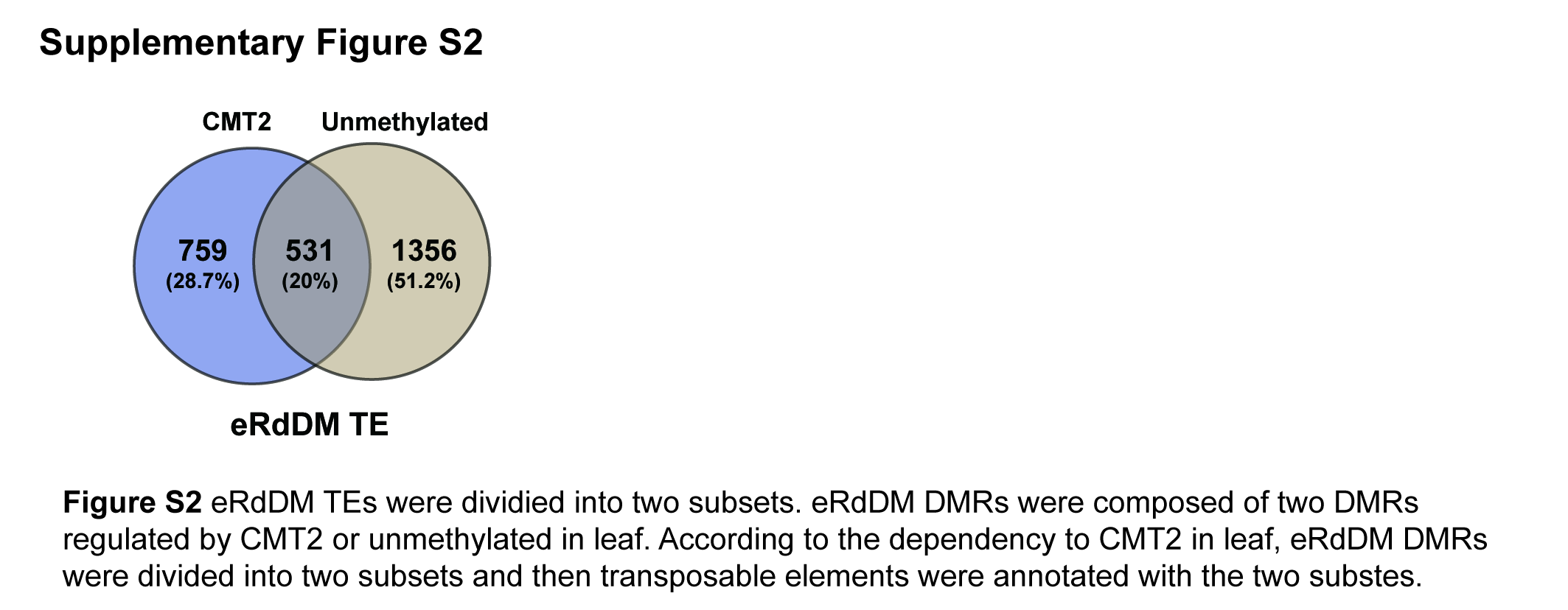

Supplement: Supplementary file 2 [file Image_2.tif]

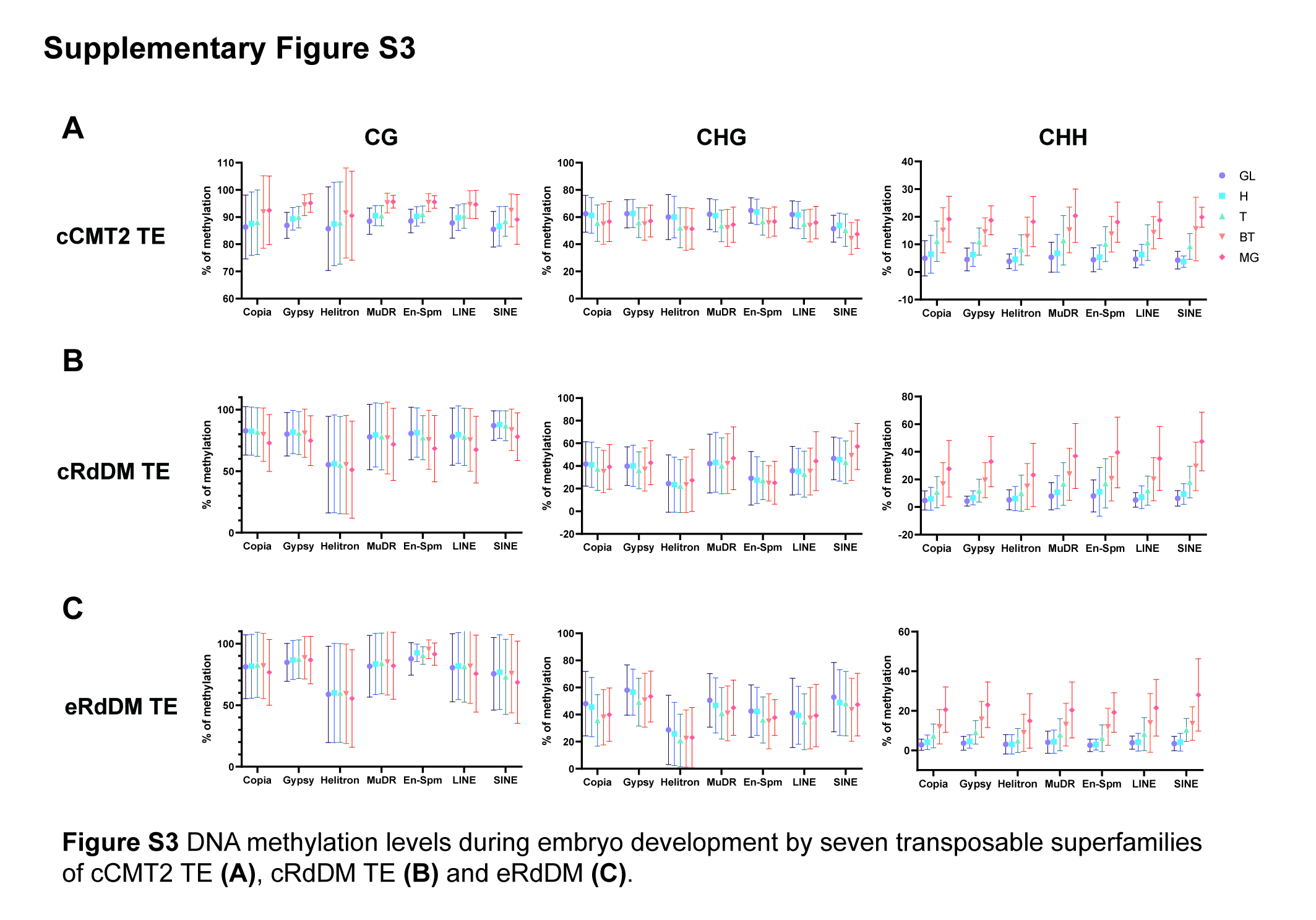

Supplement: Supplementary file 3 [file Image_3.tif]
